# Transcriptomic analysis of gene signatures associated with sickle pain

**DOI:** 10.1038/sdata.2017.51

**Published:** 2017-05-16

**Authors:** Jinny A. Paul, Anupam Aich, Juan E. Abrahante, Ying Wang, Rebecca S. LaRue, Susan K. Rathe, Krystina Kalland, Aditya Mittal, Ritu Jha, Fei Peng, David A. Largaespada, Anindya Bagchi, Kalpna Gupta

**Affiliations:** 1 Vascular Biology Center, Division of Hematology, Oncology and Transplantation, Department of Medicine, University of Minnesota, Minneapolis, Minnesota 55455, USA; 2 University of Minnesota Informatics Institute, Minneapolis, Minnesota 55455, USA; 3 Minnesota Super Computing Institute, University of Minnesota, Minneapolis, Minnesota 55455, USA; 4 Masonic Cancer Center, University of Minnesota, Minneapolis, Minnesota 55455, USA; 5 Department of Pediatrics, Division of Hematology, Oncology and Transplantations, University of Minnesota, Minneapolis, Minnesota 55455, USA; 6 Department of Genetics, Cell Biology, and Development, University of Minnesota, Minneapolis, Minnesota 55455, USA; 7 Department of Urology, University of Minnesota, Minneapolis, Minnesota 55455, USA

**Keywords:** Pain, Rat, Transcriptomics, Genetic markers, Haematological diseases

## Abstract

Pain is a hallmark feature of sickle cell disease (SCD). Recurrent and unpredictable acute pain due to vaso-oclussive crises (VOC) is unique to SCD; and can be superimposed on chronic pain. To examine the mechanisms underlying pain in SCD, we performed RNA sequencing of dorsal root ganglion (DRG) of transgenic sickle mice and their age-matched control mice expressing normal human hemoglobin A, at 2 and 5 months of age. Sickle and control mice of both ages were equally divided into hypoxia/reoxygenation (to simulate VOC) and normoxia treatment, resulting in eight groups of mice. Each group had at least six mice. RNA isolated from the DRG was sequenced and paired-end 50 bp sequencing data were generated using Illumina’s HiSeq 2000. This large dataset can serve as a resource for examining transcriptional changes in the DRG that are associated with age and hypoxia/reoxygenation associated signatures of nociceptive mechanisms underlying chronic and acute pain, respectively.

## Background and Summary

Sickle cell disease (SCD) was the first molecular disease to be identified, which occurs due to a point mutation leading to a single amino acid substitution from Glutamic acid to Valine on position 6 of the β-globin chain of hemoglobin^1^. Under low-oxygen conditions, hemoglobin with mutated β-globin polymerizes resulting in sickle shaped red blood cell (RBCs). Sickle RBCs stick and occlude capillaries leading to vaso-occlusive crises (VOCs), leading to impaired oxygen supply to the tissues, inflammation, ischemia/reperfusion injury, oxidative stress, end organ damage and severe pain. In addition to acute pain due to VOC, SCD can be accompanied by severe chronic pain which can start in infancy. Pain in SCD is considered to be worse than labor pain, often requiring hospitalization, reduced survival and poor quality of life^2^. Although both chronic and acute pain are observed in adult patients, infants and children are reported to mostly suffer from acute pain. Transition from acute to chronic pain is likely to occur during the adolescence and remains poorly understood^3,4^. Elucidating the mechanisms underlying the transition from acute to chronic pain could facilitate better pain management and targeted interventions with improved outcomes.

Dorsal root ganglion (DRG) houses neuronal cell bodies of the afferents involved in transmission of action potentials from the periphery to the spinal cord. Since, pain can start early in age in SCD, it is likely to influence the genetic architecture of the primary sensory neurons. This may lead to the hyper-excitability of spinal neurons and chronic hyperalgesia observed by us in sickle mice^5^. Thus, differences in gene expression are expected to reflect the mechanisms underlying acute and chronic pain in SCD. Therefore, we obtained the transcriptomic profile of the DRG of transgenic sickle mice under different age and pain conditions which can provide insights into the molecular changes. We chose to use HbSS-BERK sickle mice because of their similarity to human SCD with respect to pain characteristics. Here we report the transcriptomic analysis carried out among different cohorts and subsequent validation of some of the differentially expressed genes.

**Figure 1:**
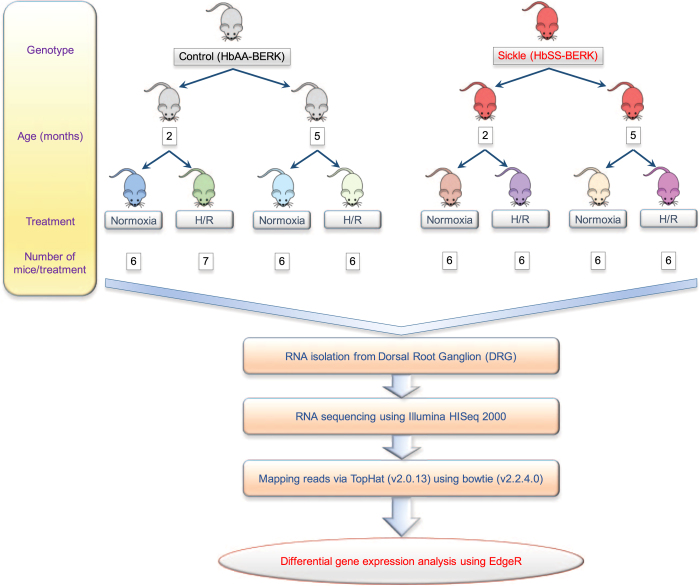
Overview of the study design. Two transgenic mouse models—HbSS-BERK (sickle) and HbAA-BERK (control) and two age groups (2 and 5 months old) of each genotype were examined. Each age group/genotype was either treated with hypoxia/reoxygenation (H/R) or treated at room air (normoxia). Each treatment was performed on at least 6 mice. RNA was isolated from the DRG of each mouse and sequenced to obtain gene profile associated with both genotypes, treatments, age and pain status. Subsequent mapping of the sequencing reads provides whole transcriptomic status of individual samples. Differential gene expression following between group comparisons is undertaken to provide insight into the genetic signatures of chronic and VOC-evoked pain. Since these sickle mice recapitulate the features of chronic pain in patients with SCD, which increases with age and the H/R evoked hyperalgesia mechanistically mimics pain due to VOC, this study design is expected to identify mechanisms involved in these sickle pain conditions.

As illustrated in the study design, two transgenic mouse models were used: [i] HbSS-BERK (sickle) mice that express human sickle hemoglobin and simulate human SCD phenotype, including pain, and [ii] HbAA-BERK (control) mice which express normal human hemoglobin A (Fig. 1). Each mouse model included two age groups: 2 months, representing young and 5 months, representing adults. These age groups were selected to understand the change in genetic signatures of the DRG with the evolution of pain, because 5 month old sickle mice show chronic pain but 2 month old sickle mice do not show significant hyperalgesia compared to control mice. Mice were further divided into two treatment groups: a) normoxia, exposure to normal room air, and b) hypoxia/reoxygenation (H/R)-treatment which represents acute pain due to VOC. These treatment conditions along with two age groups and genotypes resulted in eight different treatment groups of animals (*n*=6 in each group) qualifying the study design to evaluate the effect of age and transition from acute to chronic pain. RNA was extracted from the isolated DRG of these animals and sequenced using Illumina’s RNA-seq platform. The scope and scale of the study design will allow identification of a large array of differentially expressed genes (DEGs) that contribute to SCD pain and transition from acute to chronic pain state. This will also allow for identification of protective mechanisms/genes unknown/ not yet discovered in this disease state. We provide an example in the usage notes section to depict how this large data set can enable us to identify important genetic targets with the potential to improve the quality of life of SCD patients. Considering the comprehensive study design of this large data-set, further downstream data processing and pathway analysis will allow identification of genetic variations and associated molecular events underlying SCD pain.

## Methods

### Animals

All mice are bred in the housing at the University of Minnesota, approved by Association for the Accreditation and Assessment of Laboratory Animal Care, Int (AAALAC); and maintained under controlled environmental conditions (12 h light-to-dark cycle, at 23 °C). All experiments were performed following approved protocols from the University of Minnesota’s Institutional Animal Care and Use Committee and conform to the statutes of the Animal Welfare Act and the guidelines of the Public Health Service as issued in the Guide for the Care and Use of Laboratory Animals.

Transgenic sickle mice (HbSS-BERK) expressing >99% human sickle hemoglobin and controls (HbAA-BERK) expressing >99% normal human hemoglobin A were used^6^. HbSS-BERK mice are homozygous for knockout of both murine α and β globins and carry the linked transgenes for human α and β^S^ globins. Thus, the HbSS-BERK mice express human sickle hemoglobin, and demonstrate severe disease including reticulocytosis, hemolysis, anemia, organ damage, hyperalgesia, and a shortened life span^6–8^. The HbSS-BERK mice exhibit chronic hyperalgesia with characteristic features of sickle pain, including, mechanical, deep-tissue and thermal hyperalgesia^8^. Additionally, VOC-like pain can be evoked in this model by hypoxia/reoxygenation treatment described below. Control HbAA-BERK are bred by backcrossing HbAA-BERK with HbSS-BERK and therefore have the same mixed genetic background as HbSS-BERK, but exclusively express normal human hemoglobin A (human alpha and beta A globins) and no murine globins. The HbAA-BERK mice do not exhibit constitutive hyperalgesia.

All mice were phenotyped for sickle and normal human hemoglobin by iso-electric focusing^7^. Mouse genotypes for the transgenes for human hemoglobin A (Hba-5 Tg), human hemoglobin S (HuHbs-1 Tg), and murine α and β globins (Hba-1 KO, Hba-1 WT and Hbb-1 KO, Hbb-b1-1 WT, respectively) were determined using real time PCR (Transnetyx, Cordova, TN).

### Hypoxia/reoxygenation treatment

The mice in small rodent cages are placed in a chamber with the atmosphere controlled by a Proox Model 110 gas controller (BioSpherix, Ltd, Lacona, NY). These mice are exposed to hypoxia with 8% O_2_ and 92% N_2_ for 3 h, followed by reoxygenation for 1 h in the room air^8^. H/R treatment was repeated after one week, thus exposing the mice to two recurrent H/R treatments. DRG were collected 24 h after the second hypoxia reoxygenation treatment.

### DRG collection and RNA isolation

Mice were euthanized and DRG were collected and placed in RNA later before proceeding to RNA isolation. H/R-treated mice were sacrificed after 24 h post-2nd H/R treatment. RNA isolation was performed using TRIzol Reagent (Applied Biosystems (ABI), Foster City, CA) followed by purification using the RNeasy mini kit (Qiagen Corp., Santa Clarita, CA). The purity of RNA was checked with Nanodrop (Thermo Fisher Scientific).

### Library preparation and RNA sequencing

RNA sequencing from the collected samples was performed at the University of Minnesota Genomics Center (UMGC) using Illumina HiSeq 2000 sequencer platform. The details of the library preparation and sequencing are provided below.

#### Quality check (QC)

For the RNA samples to pass the initial quality check before preparing the sequencing library, each RNA sample needs to be more than 1 μg and must have an RNA integrity number (RIN) of 8 or greater. The total amount of RNA in each sample is quantified using a fluorimetric RiboGreen assay (R11490 Quant-iT from Thermo-Fischer) and RIN is generated for each sample by performing capillary electrophoresis using Agilent BioAnalyzer 2100.

#### Library creation

Total RNA samples are converted to Illumina sequencing libraries using Illumina’s Truseq RNA Sample Preparation Kit (Cat.# RS-122-2001). Please see www.illumina.com for a detailed list of kit contents and methods. In brief, 1 μg of total RNA from each sample is purified using oligo-dT coated magnetic beads (part of RNA prep kit). Next they are fragmented and then reverse transcribed into cDNA. The cDNA is blunt-ended, A tailed and ligated to indexed (barcoded) adaptors and amplified using 15 cycles of polymerase chain reaction (PCR) (Biorad DNA Engine Tetrad 2). Final library size distribution is validated using capillary electrophoresis and quantified using fluorimetry (PicoGreen) and via qPCR. Indexed libraries are then normalized, pooled and then size selected to 320 bp+/−5% using Caliper’s XT instrument (Perkin Elmer).

#### Cluster generation and sequencing

Truseq libraries are hybridized to a paired end flow cell and individual fragments are clonally amplified by bridge amplification on the Illumina cBot. Once clustering is complete, the flow cell is loaded on the HiSeq 2000 and sequenced using Illumina’s sequencing by synthesis (SBS) chemistry to generate 50 bp paired end reads.

#### Primary analysis and de-multiplexing

Base call (.bcl) files for each cycle of sequencing are generated by Illumina Real Time Analysis (RTA) software during the sequencing run on HiSeq 2000. Primary analysis and de-multiplexing are performed using Illumina’s CASAVA software 1.8.2. The end result of the CASAVA workflow is de-multiplexed FastQ files that are subjected to subsequent analysis described below. The CASAVA software assigns quality scores to the FastQ files.

### Alignment of the reads and differential gene expression (DGE) analysis

The Illumina read files were processed via a pipeline developed by the University of Minnesota Informatics Institute (UMII) in collaboration with the Minnesota Supercomputing Institute (MSI) and the UMGC. Briefly, FastQ files were trimmed via trimmomatic using the following parameters: -phred33 -threads 8, ILLUMINACLIP, LEADING:3 TRAILING:3 SLIDINGWINDOW:4:16 MINLEN:25. After trimming, mapping was performed via TopHAT (v2.0.13) using bowtie (v2.2.4.0). This mapping results in output of one binary alignment/map (BAM) file for each sample matched to a reference mouse genome. Finally, fragments per kilobase of transcript per million mapped reads (FPKM) values were calculated as expression data via cuffquant (--quiet -u --max-bundle-frags 10,000,000 and cuffnorm). ‘featureCounts’ was used to count the reads. The primary output files from the analysis pipeline are FastQC files, raw count expression tables as text files and expression data as FPKM values.

Most DGE analysis platforms use raw read counts as inputs. We performed DGE analysis among different treatment groups using CLC genomics workbench (CLCGWB v 9.0.1, Qiagen Bioinformatics; license available through MSI) which utilizes a platform called empirical analysis of DGE in R (edgeR) from Bioconductor. EdgeR performs the statistical analysis of raw counts from high-throughput sequencing of multi-factorial comparative experimental designs. This platform exploits the advantage of using negative binomial distribution to model the transcript raw counts so that the biological variation is distinguished from technical variations. In other words, the variability within each of the two groups in comparison are taken into account assuming that the dispersion in each group is identical, which is usually not true in case of original data. As EdgeR separates technical variance from biological variance, DGE can be reliably estimated between groups with small number of samples. We imported the raw read count files from two groups of interest into the CLCGWB interface to analyze the DGE of each such comparison. The resultant file contains a list of genes that are differentially expressed in these two groups with their statistically significant *P* values for both false discovery rate (FDR) and Bonferroni corrections. We chose Bonferroni corrections (*P*<0.05) to be our selection criteria which provides more stringent statistical significance for the data. For our analyses, we selected the genes with equal to or greater than two-fold changes in the comparison with this Bonferroni correction criteria.

### Real time quantitative polymerase chain reaction (RT-qPCR)

To validate the expressions of the genes of interest obtained from our DGE analyses, we independently collected samples from sickle mice and matched controls. For validation experiments, RNA was isolated and the purity of the RNA was checked as mentioned above. Thereafter, up to 1 μg of RNA was converted into cDNA by using a high capacity RNA to cDNA kit (Applied Biosystems, cat# 4387406) and the resultant cDNA was diluted 20-fold to be used in q-PCR reaction mix. Quantifications of RNA expression levels were performed in an Applied Biosystems 7000 RT-PCR system. GAPDH was used for normalization. The total volume of reaction mixture was 25 μl containing 12.5 μl Power Sybr Green PCR master mix (Applied Biosystems, cat# 4367659), 2 μl of diluted cDNA template, 0.1 μl of sense primer, 0.1 μl of anti-sense primer and 10.3 μl of distilled water. All the primers were synthesized by Integrated DNA Technologies (IDT). Thereafter, the calculations of relative gene expression were done using the 2(-Delta Delta C(T)) method^9^. The PCR primers were as follows:

Small proline rich protein 1A (Sprr1a): Sense: 5′- GCT GTC TAT CCT GCT TAT GAG TC-3′; Anti-sense: 5′- CTT TGG GCA ATG TTA AGA GGC-3′

SerpinA1e: Sense: 5′- CCC TAT CTT GCA CTT CAA CCG-3′; Anti-sense: 5′- CAG CCC GTG TTT AAT GGA AA-3′

SerpinA3g: Sense: 5′- GTG TCG GAT GTT GTG CAG TT-3′; Anti-sense: 5′- CAC CAT TTG GGA AGT TCA T-3′

Glyceraldehyde 3-phosphate dehydrogenase (GAPDH): Sense: 5′- ATG TGT CCG TCG TGG ATT TGA-3′; Anti-sense: 5′- ATG CCT GCT CTA CCA CCT TCT T-3′

## Data Records

RNA-seq data files in FastQ format were deposited in the Gene Expression Omnibus (GEO, NCBI) under the accession number GSE86418 (Data Citation 1). This accession contains a total of 98 FastQ files resulting from the forward and reverse paired-end runs for each of the 49 samples. The FastQ format data serves as the raw data from the sequencing, which are subjected to further downstream processing. Additionally, the ISA-tab file provided with this publication includes detailed metadata for the study and standardized representation of the study design. Examples of processed data in two .xlsx files obtained from UMII-MSI-UMGC analysis pipeline are also stored in this accession: a) Raw_Read_Count_all samples.xlsx and b) FPKM_Values_all samples.xlsx. As the names suggest they contain the raw read counts and FPKM expression values obtained from this data. The metadata for these files are also presented in ISA-tab file provided with this publication. Three excel files named ‘edgeR_Age_Comparison_Output files’, ‘edgeR_Genotype_Comparison_Output files’ and ‘edgeR_Treatment_Comparison_Output files’ have been provided in figshare with full outputs from edgeR DGE analysis (Data Citation 2).

## Technical Validation

In this study, best practices of NGS analysis were used to assess the quality of the reads and align them against a reference genome as described in the methods section. FastQ files were fed into the UMII-MSI-UMGC analysis pipeline that generates information on the mapping and gene quantification of the reads.

**Figure 2:**
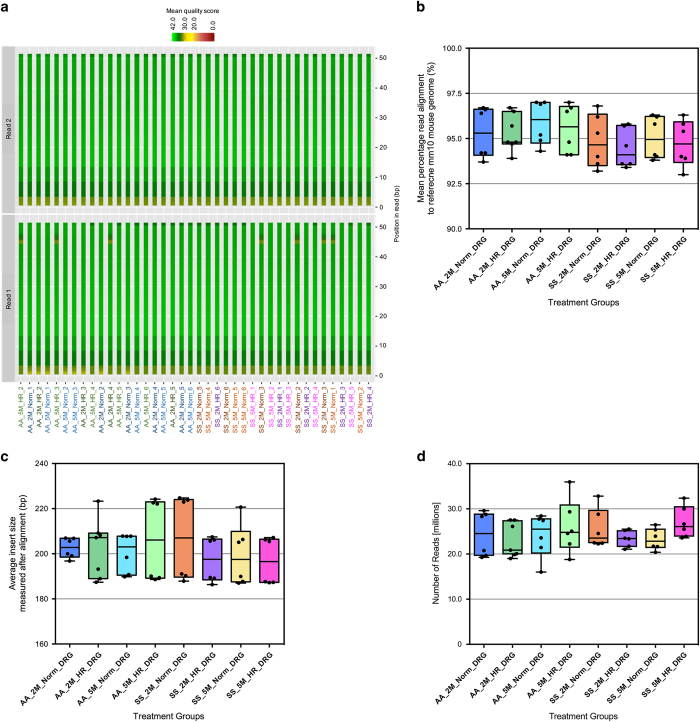
Robustness and quality of RNA sequencing run and processed data. Each sub-figure illustrates the results for the following quality metric: (**a**) read quality of both runs in paired-end sequencing based on Phred quality score, (**b**) percentage of reads aligned to UCSC mm10 mouse reference genome, (**c**) average insert length in base pairs measured after alignment, and (**d**) number of reads in each paired-end runs. Each box plot displays the range and distribution of a quality metric computed for sequencing sample runs. The horizontal line is the median, the top of the box is the upper quartile, and the bottom of the box is the lower quartile.

The representative quality plots from the filtered fastQ files of this data-set are shown in Fig. 2a. It demonstrates that the accuracy of the base calling in these sequences are of the highest quality as most of the nucleotides in each of the reads contain Phred score exceeding 30 and close to 40 (the highest score). Additionally, when we mapped the sequence reads with the reference mouse genome mm10, all of the eight (8) sample groups had mean percentage of mapping near 95% with a very small s.d. of ~1–2% (Fig. 2b). Samples’ integrity and purity was near optimal conditions as reflected by the small amounts of contamination and other quality issues that may have arisen from the sequencing library preparation from the extracted RNA. The average insert size for each sample group presented in Fig. 2c also serves as another quality check as the error in the fragmentation chemistry and PCR amplification during library preparation may introduce improperly sized fragments in the sequencing library. The low variation of the average insert size measured after the alignment in all the groups reflects the accuracy of the library preparation and also eliminates the chances of unusual raw counts from a non-uniform sequencing library.

Another quality control measure of the data originates from the fact that the sequencing read depths used in this study ranged from 20–30 millions [Fig. 2d]. These high read depths coupled with the fact that the sequencing was done in paired-end fashion ensured the unambiguous placement of the repetitive regions^10^. The large number of biological replicates (6) for each group along with high read depth enhanced the statistical power of these dataset and resulted in a very low coefficient of variance^11^. Consequently, the number of false positives expected during the differential gene expression promises to provide specific variations even with small fluctuations at the transcript level^11^. Therefore, robustness of this dataset is reflected from the above parameters and the DGE analysis performed using this dataset will be prone to error at the least possible level and the results should be of high accuracy and power.

## Usage Notes

SCD patients suffer from life-disabling acute and chronic pain, and management of such pain requires molecular level understanding of the pain mechanisms originating from the pathophysiology of the disease itself. To the best of our knowledge this is the first of its kind gene expression dataset generated from an experimental design with a focus on the transition from acute to chronic pain and the VOC evoked pain in SCD. Analysis of DEGs from multiple possible sets of comparisons, provide the possibility of specifying the roles of such genetic markers in mechanistic pathways leading to the identification of treatable targets for pain in SCD, and for other chronic pain conditions. We performed mapping of this dataset via TopHat using Bowtie to the reference genome mm10. Several other platforms such as STAR^12^, MapSplice^13^, Cufflinks^14^ and others^15^ can be used to align and map reads from this dataset. Downstream quantification of transcript abundance can be performed using multiple platforms^16^, of which, we used cufflinks in our analysis. From the counts of the raw reads of the expression values or FPKM values, DGE analysis can be executed using a platform of choice from an array of developed packages such as DESeq2 (ref. 17), Cuffdiff^18^, edgeR^19^ and many more. Here we present a snapshot of analysis of DEGs that can be performed using this dataset and we provide RT-qPCR validation for three genes that we found to be differentially expressed in our analysis.

We used edgeR from CLCGWB software package for performing DGE analysis from the raw read counts obtained after TopHat-Bowtie mapping and subsequent quantification of the gene expression. From the set of 8 groups of samples, if we compare two (2) groups at a time, we can have a total of 56 (^8^P_2_=56) groups of comparisons. From these 56 groups, there will be a much lesser number of comparisons which are of meaningful interpretation. The DGE analysis was performed using edgeR as this platform applies a normalization of the raw read counts which ensures that the genes with low abundance but highly variable read counts will have same variance as the genes with high abundance. Therefore, while edgeR provides more strict bounds for the calculations of DGE, it also enables decoupling of the technical and biological variances, even with small set of replicates, enhancing the overall statistical power of the comparisons.

**Figure 3:**
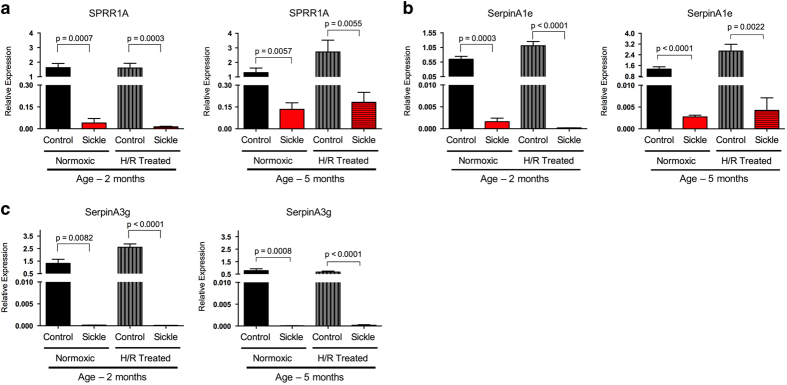
RT-qPCR validation and example of identification of genetic markers. The RT-qPCR expression data provides validation for genes that were found to be differentially expressed in the RNA-seq data at two different ages of sickle and control mice under two different treatment conditions. (**a**), (**b**) and (**c**) show qPCR validation for differentially expressed SPRR1A, SerpinA1e and SerpinA3g genes, respectively.

As an example of the reliability of this dataset, here we present a RT-qPCR validation of three genes which were identified from the edgeR DGE analysis: Sprr1a, SerpinA1e and SerpinA3g. The comparison groups and corresponding differential expression values of these three genes are presented in Table 1, and the validation with qPCR is presented in Fig. 3a–c. The comparison groups were chosen to identify the DEGs that differ under the influence of the different genotypes at the same age and same treatment conditions, thereby, reflecting the effect of the disease itself. We chose the Bonferroni correction for the statistical analysis from the edgeR analysis and filtered for the genes that had at least two fold changes in the comparisons. In Table 1 we observe that all these genes are down-regulated in the diseased animal compared to control animal, when there is a differential expression. This type of analysis could reflect the genetic signatures that are inherent to the disease itself, rather than being effects of environmental factors or age. We can see from the qPCR validation of these genes (Fig. 3a–c) that indeed the effect of the SCD genotype are reflected significantly in these data as the genes remain significantly down-regulated in the SCD groups compared to age-matched and treatment-matched control groups. These data provide a comprehensive resource to identify genetic-markers and signatures specific to chronic and VOC-evoked pain in SCD, and plausibly in other pain conditions.

## Additional information

**How to cite this article**: Paul, J. A. *et al.* Transcriptomic analysis of gene signatures associated with sickle pain. *Sci. Data* 4:170051 doi: 10.1038/sdata.2017.51 (2017).

**Publisher**’**s note**: Springer Nature remains neutral with regard to jurisdictional claims in published maps and institutional affiliations.

## Supplementary Material



## Figures and Tables

**Table 1:** Fold changes in expression values (obtained from edgeR analysis of RNA-seq data) of the selected genes in four comparison groups reflecting the effect of genotype (i.e, the sickle cell disease itself).

	**AA_2M_Norm versus SS_2M_Norm**	**AA_2M_HR versus SS_2M_HR**	**AA_5M_Norm versus SS_5M_Norm**	**AA_5M_HR versus SS_5M_HR**
Sprr1a	ND	ND	ND	−63.97
SerpinA1e	ND	−21.43	−27.93	ND
SerpinA3g	ND	−1.94	−1.97	−2.00
Abbreviations: AA and SS represents control HbAA-BERK and sickle HbSS-BERK mice, respectively; 2M and 5M refer to 2 months old and 5 months old mice, respectively; HR and Norm represent hypoxia/reoxygenation treated and untreated normoxic mice, respectively. Thus, for example, SS_2M_HR represents hypoxia/reoxygenation treated 2 months old sickle transgenic mice. ND, no differential expression when Bonferroni statistical corrections are employed.				
Note that that SS groups have downregulated expression of the specific gene compared to the respective AA mice.				
